# Strengthening the Immunization System Through Private Provider Engagement to Improve Vaccine Uptake in Urban Settlements of Karachi, Pakistan: A Before–After Study

**DOI:** 10.3390/vaccines14030205

**Published:** 2026-02-26

**Authors:** Zahid Memon, Ammarah Ali, Shifa Habib, Wardah Ahmed, Fizza Ansar, Maheen Kalwar, Iqbal Azam, Lala Aftab, Ahsanullah Bhurgri, Shehla Zaidi

**Affiliations:** 1 Department of Community Health Sciences, Aga Khan University, Stadium Road, Karachi 74800, Pakistan; ammarah.ali@aku.edu (A.A.); shifa.habib@aku.edu (S.H.); ahmed.wardah@aku.edu (W.A.); fizza.ansar2@aku.edu (F.A.); maheen.kalwar@aku.edu (M.K.); iqbal.azam@aku.edu (I.A.); lala.aftab@aku.edu (L.A.); shehla.zaidi@aku.edu (S.Z.); 2 Expanded Program on Immunization, Department of Health, Government of Sindh, Karachi 74200, Pakistan; ahsanullah@rizconsulting.biz

**Keywords:** childhood vaccination, immunization coverage, vaccine uptake determinants, private sector engagement, integrated services, Pakistan, urban

## Abstract

Background: We aimed to evaluate the impact of a Private Provider Engagement (PPE) model that integrated neighborhood private health providers into the immunization system to improve vaccine uptake and reduce coverage disparities among marginalized communities in Karachi, Pakistan, where health inequities and the risk of vaccine-preventable diseases remain high. Methods: Routine immunization service corners were established at nine private clinics in urban settlements of eight high-risk union councils (HRUCs) in Karachi. A quasi-experimental before-and-after study design was used with a baseline survey conducted in May–July 2022 and an end-line survey in April–June 2024. Households were selected using a multistage cluster sampling approach, and data were collected from parents or primary caregivers of children aged 4–11 months residing in the catchment areas for >3 months, using an adapted WHO immunization coverage questionnaire. The primary outcome was child immunization status for BCG, Polio, Pentavalent (DTP-3), Rotavirus, PCV, TCV, and MR vaccines, categorized as fully vaccinated, partially vaccinated, or unvaccinated, and verified through vaccination cards or caregiver recall. Multinomial and binary logistic regression were used to investigate factors associated with immunization coverage. Results: A total of 2167 children were surveyed (1141 children at baseline; 1026 children at end-line). The proportion of fully immunized children more than doubled across sexes, with significantly higher adjusted odds at endline (aOR: 6.34, 95%CI: 2.45–16.21). Age-appropriate uptake of all antigens improved, with over fourfold odds for receiving the Penta-3 vaccine (aOR 4.55, 95%CI: 3.55–5.82) and more than threefold odds for receiving the MR-1 Vaccine (aOR 3.67, 95%CI: 2.37–5.67). Parental education strongly predicted immunization, with the highest odds among children of fathers with secondary or higher education or skilled labor. Fully immunized Pashto-speaking children increased at endline but had the lowest odds compared to Urdu-speaking children. Conclusion: The PPE model increased vaccination coverage and reduced disparities in Karachi’s urban settlements, demonstrating potential for scale-up to strengthen routine immunization and reduce the number of zero-dose children.

## 1. Introduction

Immunization is a cornerstone of primary health care and one of the finest cost-effective health investments, strengthening health systems by preventing illnesses and infectious disease outbreaks. Achieving high immunization coverage is therefore a global imperative. Between 2020 and 2030, vaccines are projected to save 28 million children younger than 5 years and avert 1.8 billion disability adjusted life years (DALYs) in this age group [1]. Despite this progress, 25 million infants missed age-appropriate vaccinations in 2021, with five countries, including Nigeria, India, the Democratic Republic of Congo, Pakistan, and Ethiopia, accounting for over half of all zero-dose children globally [2].

Globally, addressing vaccine inequities has become a central priority. The Immunization Agenda 2030 (IA2030) provides a strategic framework to ensure that everyone, everywhere, at every age fully benefits from vaccines. It also puts a specific emphasis on reaching marginalized communities in urban slums, as a substantial proportion of missed immunizations can be attributed to the “urban paradox”. Although nearly 58% of the world’s population now lives in cities [3], the benefits of urbanization, including improved access to services, remain unevenly distributed. In many low- and middle-income countries (LMICs), including Pakistan, rapid urban growth is concentrated in informal settlements and peri-urban slums, where health inequities are stark and routine immunization delivery remains chronically constrained [4].

In Sindh province, the proportion of children who were immunized up to the first dose of Measles Rubella antigen (MR1) was reported at 61.1% in 2023, with urban areas showing comparatively higher coverage in Karachi (65.4%) and Hyderabad (67.9%) [5]. However, the urban slums and settlements of Karachi depict a different picture with immunization coverage for MR1 reported at 54% and for Pentavalent-3 at 30.8% [6,7]. These persistent gaps are particularly concerning in Karachi’s densely populated urban settlements, home to Afghan refugees and internal migrants, and characterized by inadequate health infrastructure and high population mobility. These underserved communities represent hotspots of critically low immunization coverage, undermining progress towards equitable vaccine access and the broader control of polio and other vaccine-preventable diseases [8,9,10,11].

In 2014, the World Health Organization (WHO) recognized children in marginalized urban communities as a key population requiring targeted intervention [4]. However, prior studies from Pakistan have highlighted persistent barriers despite sustained efforts, including high rates of vaccine refusal, low routine coverage, and geographical disparities in uptake [12,13]. While vaccine supply and safety have improved, major gaps persist in outreach, supervision, and client communication [14]. At the household level, mothers and caregivers commonly report lack of awareness, fear of side effects, and long distances to clinics [10,15]. At the systems level, weak governance, fragmented management, and low community trust hinder coverage [8,11].

In response, an implementation research pilot is being jointly conducted by the Expanded Programme on Immunization (EPI), Department of Health, Government of Sindh, and the Aga Khan University to address vaccine inequities in Karachi’s squatter settlements (Supplementary Material Figure S1).

Private Provider Engagement (PPE) Model: This initiative engages neighborhood private healthcare providers to extend immunization services, nutritional screenings, and health education across eight high-risk union councils (HRUCs) of Karachi in this study. By integrating private-sector health facilities, the PPE model sought to address the limitations of government-run primary healthcare (PHC) systems in urban settings, as private providers are often preferred and more accessible sources of health care in these areas but typically lack routine immunization and other preventive services [14,16,17].

This study, part of the larger project, covered catchment areas of nine centers in eight HRUCs. The aim was to assess the effect of the PPE model on vaccination status and coverage among children aged 4–11 months. Vaccination coverage and status by sociodemographic factors were measured. Findings will be utilized to design contextualized strategies to address immunization gaps in marginalized urban communities in Karachi and similar settings in Pakistan, and are presented here for the benefit of the larger academic and development communities.

## 2. Materials and Methods

Study design and setting: This quasi-experimental before-and-after study was conducted in Karachi, Pakistan, a city with a population of 20 million, administratively divided into seven districts and 178 union councils (UCs). UCs are the smallest administrative units responsible for public service delivery. Among the 39 UCs under polio transmission surveillance in Karachi, eight are categorized as Super High Risk Union Councils (SHRUCs) while an additional 31 are classified as HRUCs. The study was conducted in urban settlements within eight HRUCs, covering nine polygons, comprising a private provider center covering an approximate catchment population of at least 25,000. Baseline and endline surveys were conducted between May to July 2022 (before) and April to June 2024 (after). The study areas represent typical low-income settlements with multiethnic populations, low literacy rates, and poor living conditions.

Key Components of the PPE Model

1. Private Provider Engagement: Routine immunization service corners were established at nine private provider clinics (including for-profit and non-profit), selected based on zero-dose hotspots, distance from public health facilities, licensed providers’ and their willingness to offer free immunization services. Each site was staffed with two vaccinators and one female counsellor, offering vaccination, nutrition screening, and counselling on breastfeeding, hygiene, and child nutrition. Operations and HR were supported through a third-party payment mechanism, with regular monitoring by Sindh EPI. The vaccines, cold chain equipment, reporting tools and training materials were provided by EPI.
2. Community Hosted Engagement Events: Local community hosted health events focusing on—social get together, routine Immunization, Nutrition Screening, and Counseling. The events are named in local terms as “Health Mehfil” if organized at the venue of birth attendants, schools, and madrassas (religious schools), and “Health Baithak” if organized at the house of community influencers called “Autaaq” and “baithaks”. Birth attendants were trained to refer newborns, improving the timely uptake of first doses.
3. Digital Communication: Immunization information was disseminated via WhatsApp broadcasts and digital marketing, enabling private providers to promote services and enhance community awareness.
4. Nutrition Screening and Counselling: Routine and event-based screening for children under 5 years was conducted, with referrals of severe acute malnutrition cases to outpatient therapeutic program (OTP) sites.

Study population: Eligible participants were parents or primary caregivers of children aged 4–11 months who had resided in the study polygon for at least three months. Either parent (mother or father) was eligible to participate, and in their absence, the child’s routine caregiver was interviewed.

Operational definitions: In this study, we define the following terms:

- Private Provider Engagement Model: Establishment of EPI centers at local neighborhood private providers formally integrated into the EPI system to provide routine immunization services, using government-supplied vaccines, standardized recording/reporting tools, and referral mechanisms.
- A fully immunized child is a child who has received all vaccine doses due at their current age as per the Pakistan EPI schedule [18,19].
- A zero-dose child, as defined by the Global Alliance for Vaccines and Immunization (GAVI), has not received the first dose of the pentavalent vaccine (Penta-1) [20].
- A partially vaccinated child has missed one or more routine immunizations previously as per the EPI schedule.
- An unvaccinated child has failed to receive any routine vaccination.
- The variable unskilled refers to individuals who have not acquired any specialized or technical training for employment [21]. This category in our study also includes unemployed individuals, such as housewives.
- Skilled providers include individuals engaged in technical occupations that require formal training [21].

Sample size: The study was powered (at least 80%) to detect at least a 10% percent increase in Penta-3 coverage over two years. Based on a baseline Penta-3 coverage of 68% among infants in Karachi from the Multiple Indicator Cluster Survey (MICS) of Sindh 2019 [22], using a two-sided 5% significance level, and a minimum design effect of 3, the required sample size was calculated to be 950 children aged 4–11 months in this group for each survey. During the surveys, 1141 children were enrolled at baseline and 1026 at endline in this group.

Sampling technique: A multistage cluster sampling approach was applied. Each cluster comprised seven eligible households. In the first stage, the clusters were determined at the UC level using the probability proportional to size method, and the same methodology was applied at the polygon level. Within clusters, the first household was randomly selected by pen spin, while subsequent households were identified by checking every third household for eligibility until the cluster was completed. If a household was ineligible, the next was checked, and a new direction was chosen for each cluster.

Data collection: A pre-tested, close-ended digital questionnaire adapted from the WHO immunization coverage tool was used to collect data on immunization status, sociodemographic characteristics, and health-seeking behavior. After explaining the research purpose and obtaining written informed consent, parents or caregivers were interviewed in person by trained local research assistants using Android tablets with the open-source EpiCollect platform. To ensure data quality, 10% of households were randomly reinterviewed, and spot checks were conducted throughout data collection.

Statistical analysis: Data were analysed using Stata version 17.0. Descriptive statistics, including frequencies, percentages, means, and standard deviations (SDs), were calculated to summarize demographic and socioeconomic characteristics of children and their caregivers. Immunization status categorized as fully immunized, partially immunized, and unvaccinated, was cross tabulated against sociodemographic variables at baseline and endline. Prevalence estimates and 95% confidence intervals (95% CI), were used to describe the change in average coverage of vaccines between the two survey time points. Binary Logistic regression model was used to calculate adjusted odds ratios (ORs) and 95% confidence intervals (CIs) for age-appropriate antigen uptake from baseline (reference) to endline among children, where each antigen (e.g., BCG, OPV0, OPV1, etc.) was treated as a binary outcome, and each model was adjusted for survey time (baseline vs. endline), child’s sex, age (in days), maternal and paternal education, maternal and paternal occupation, and ethnicity. The multinomial logistic regression models were used to model the probability of being fully or partially immunized relative to being unvaccinated as the reference, while adjusting for the same covariates. The proportional odds assumption was formally tested and found to be violated for several covariates, indicating that the effect of predictors was not constant across ordered categories. Therefore, multinomial logistic regression models were used. A design-based model was used, and cluster adjustment was performed in both multinomial and binary logistic models. All *p*-values reported have statistical significance defined at *p*-values < 0.05.

Ethical considerations: The study received approval from the Ethical Review Committee (ERC) of Aga Khan University, Karachi, Pakistan (ERC number 2022-7079-20320). Participation in the survey was voluntary, and written informed consent was obtained from all participants. Data were encrypted, password protected, and anonymized using unique identifiers.

This study is reported in accordance with the TREND (Transparent Reporting of Evaluations with Non-randomized Designs) guidelines.

## 3. Results

Of the 2167 children aged 4–11 months included in the study, 1141 were surveyed prior to the introduction of the PPE model and 1026 afterwards, from the catchment areas of nine established EPI centres in Karachi. Vaccination status was validated through immunisation cards or caregiver recall. The gender distribution was nearly equal in both surveys, approximately 50% (Table 1).

Vaccination status by sociodemographic characteristics: The proportion of fully immunized children more than doubled in both sexes, increasing from 24.25% to 51.05% among males and from 20.31% to 51.69% among females from baseline to endline. Correspondingly, the proportion of partially immunized children declined from 72.57% to 47.80% in males and from 77.26% to 47.71% in females (Table 1). The proportion of unvaccinated children also decreased, from 3.19% to 1.15% in males and from 2.43% to 0.60% in females.

Improvements were also observed across all parental education and occupation categories. Although most mothers had no formal education, this proportion decreased from 63.8% at baseline to 59.45% at endline. Among these mothers, 13.32% of children were fully immunized and 82.83% were partially immunized at baseline, compared with 42.79% being fully immunized and 55.90% being partially immunized at endline. Similarly, among the 55.30% fathers with no formal schooling at baseline, the proportion of completely immunized children (12.04%) was significantly lower than that of partially immunized children (83.36%). At endline, comparable proportions of complete (43.93%) and partial (54.72%) immunization were noted among the 50.58% of fathers with no formal schooling. A greater proportion of parents had secondary or higher education than only primary education in both surveys, with higher partial immunization observed at baseline and higher complete immunization observed at endline.

Regarding occupation, mothers predominantly reported engagement in unskilled work or domestic roles, although this proportion decreased from 97.02% at baseline to 91.72% at endline. Across both the maternal employment categories, a higher proportion of children were partially immunized than fully immunized at baseline; however, at endline, the proportion of fully immunized children exceeded that of partially immunized children. Among fathers, unskilled or informal employment accounted for 59.07% at baseline, whereas skilled employment increased to 70.66% at endline. Correspondingly, among children of skilled fathers, full immunization coverage was lower than partial immunization at baseline (30.62% vs. 68.31%) but higher at endline (52.83% vs. 46.48%).

Ethnic composition remained relatively consistent, with Pashto-speaking families comprising approximately one-third of respondents in both surveys. Urdu-speaking families were the second most prevalent group, followed by Saraiki- and Sindhi-speaking families, while Baluchi households constituted the smallest proportion. At baseline, partial immunization exceeded full immunization across all ethnic groups. At endline, Urdu-speaking, Baluchi, and Punjabi groups demonstrated higher proportions of fully immunized children compared with partially immunized children. Although Pashto-speaking children had the lowest full immunization coverage at both baseline (11.27%) and endline (43.19%), they exhibited the highest partial immunization coverage in both surveys (83.94% and 55.64%, respectively).

Total antigen coverage and changes in the vaccination coverage between baseline and endline are represented in Table 2.

Impact of PPE model on vaccination status Multinomial logistic regression (Table 3), using the unvaccinated group as the reference, indicated that the odds of complete immunization at endline were significantly higher compared with baseline (aOR: 6.34, 95% CI: 2.45–16.21). Adjusted odds of partial immunization showed no significant change (aOR: 1.64, 95% CI: 0.64–4.14).

Sociodemographic predictors of full and partial immunization: Several sociodemographic factors were strongly related to a child receiving all age-appropriate vaccinations at the univariate level, including the survey period, parents’ education and occupation, and household ethnic background (Table 3). In multivariable analysis, only a subset of these variables retained independent significance. Time of survey emerged as the strongest predictor, with children surveyed at endline having substantially higher odds of receiving all antigens due at their age (aOR 6.34, CI 95% 2.45–16.21), indicating the cumulative effect of the PPE intervention over time. Fathers with secondary or higher education had nearly eight times higher odds of fully immunizing their children (aOR 7.82, 95% CI: 2.09–29.24). Occupation showed minimal adjusted impact, though children of skilled fathers were more likely to be fully (aOR: 2.92, 95% CI: 1.38–6.18) as well as partially immunized (aOR: 2.29, 95% CI: 1.09–4.81). Among ethnic groups, compared with Urdu-speaking households (reference), Pashto-speaking households had significantly lower odds of full immunization (aOR 0.06, 95% CI: 0.05–0.82), followed by Balochi-speaking households (aOR: 0.07, 95% CI: 0.04–1.42).

**Figure 1:**
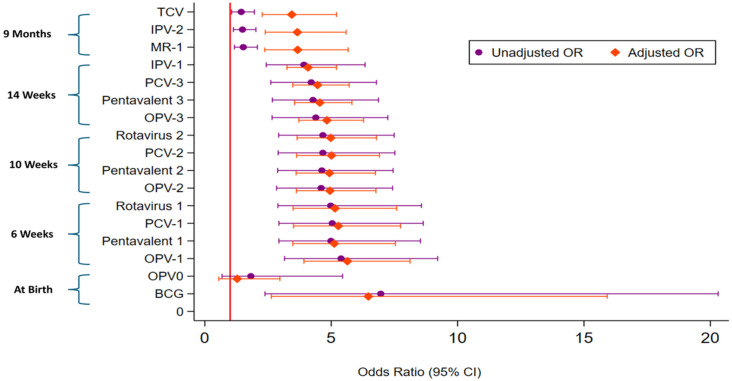
Forest plot showing Binary Logistic Regression Results for Age-Appropriate Antigen Uptake from Baseline to Endline Among Children Aged 4–11 Months. **Notes:** Unadjusted odds ratio (OR) and 95% confidence interval (CI): Purple circles (●) and lines Adjusted odds ratio (OR) and 95% confidence interval (CI): Orange diamonds (◆) and lines The vertical red line at OR = 1 represents the null value (no difference in odds). Adjusted models controlled for child’s sex, age (days), maternal and paternal education, maternal and paternal occupation, and ethnicity.

Impact of PPE model on antigen-specific coverage: Overall, the PPE model substantially improved antigen uptake between baseline and endline as mentioned in Table 2. After adjusting for covariates (Figure 1), the red vertical line represents the null value (odds ratio = 1), indicating no association between vaccination and the outcome, significant improvements were observed for all primary series vaccines including; BCG (aOR 6.47, 95% CI: 2.63–15.92), OPV-1 (aOR 5.64, 95% CI: 3.92–8.12), Penta-1 (aOR 5.12, 95% CI: 3.48–7.54), PCV-1 (aOR 5.28, 95% CI 3.51–7.74), and Rota-1 (aOR 5.15, 95% CI: 3.49–7.59), compared to baseline. Subsequent doses also improved significantly, such as Penta-3 (aOR 4.55, 95% CI: 3.55–5.82), PCV-3 (aOR 4.46, 95% CI: 3.48–5.71), and OPV-3 (aOR 4.83, 95% CI: 3.72–6.28). Vaccines administered at 9 months also showed significant increases, although the effect size was comparatively smaller (MR-1: aOR 3.67, 95% CI 2.37–5.67; IPV-2: aOR 3.66, 95% CI 2.39–5.59; TCV: aOR 3.44, 95% CI 2.27–5.21). However, OPV0 showed only a minimal increase from baseline (aOR: 1.28, 95% CI: 0.55–2.97).

## 4. Discussion

Our study demonstrates a substantial improvement in vaccination uptake among children aged 4–11 months in the urban HRUCs of Karachi following the introduction of the PPE model. The proportion of fully immunized children more than doubled across both sexes over the intervention period, with corresponding reductions in partial and non-immunization. Importantly, gains were observed across all antigens and sociodemographic strata, with marked improvements among children of parents with no formal education, those from households engaged in unskilled labor, and those belonging to traditionally under served ethnic minority groups such as Pashtun or Balochi. The results indicate that the PPE model not only improved overall immunization coverage but also helped narrow existing equity gaps in immunization.

The findings from this study align with global immunization frameworks and contribute to achieving IA2030 targets. The observed reduction in zero-dose children and the substantial increase in full immunization coverage directly support IA2030’s Impact Goal 2, which aims to promote equity through a 50% reduction in zero-dose children by 2030. Furthermore, the PPE model’s success in reaching marginalized ethnic groups and socioeconomically disadvantaged households demonstrates how local innovations can operationalize global strategies.

The private health sector is increasingly recognized as a critical partner in supplementing public health system [23]. The need for such engagement is underscored by the reliance on private providers in urban areas and the scarcity of public health infrastructure. Global evidence indicates that private-sector engagement can expand immunization coverage, particularly among zero-dose and under-immunized children in marginalized settings. For instance, partnerships with non-governmental organizations and private facilities in Bangladesh, Sudan, Nigeria, India, and Angola have demonstrated significant gains in full immunization coverage [24,25,26,27,28]. Consistent with these findings, our results showed that the proportion of zero-dose children decreased, FIC nearly doubled, and the largest improvements were seen among children of uneducated parents, unskilled labor households, and minority ethnic groups. Together, these findings reinforce that private-sector engagement can increase service reach while reducing socioeconomic and geographic inequities in vaccine uptake [14,24].

Consistent with prior literature, parental education above the secondary level was related with higher immunization coverage [29,30,31]. These findings advocate for targeted, goal-directed educational interventions to raise vaccine awareness and acceptance among less educated populations, alongside intensified governmental efforts to ensure universal access to primary education [31]. Parental education and community engagement may also serve as enablers of vaccination card retention, a finding supported both by our data and existing studies [29,32].

Our study highlights that Urdu-speaking families were the most receptive to immunization. However, the stratified analysis by ethnicity revealed, Pashtun families showed a profound relative improvement following implementation of the PPE model, despite having the lowest baseline coverage. This pattern suggests that the intervention may have mitigated longstanding dissatisfaction and mistrust in public health services [33,34]. These finding reflects persistent gaps in culturally responsive intervention design and service delivery in Karachi’s slums, where heterogeneous populations are exposed to conservative beliefs, myths, and misinformation [35,36]. Moreover, our results further emphasize the importance of contextually tailored community engagement strategies such as engaging male family members, community leaders, and religious figures through community spaces such as health mehfils and baithaks proved valuable for building trust and generating vaccine demand in communities where women possess limited decision-making autonomy [30,37].

Despite immunization been traditionally provided by public sector, initiatives that engage private providers have shown marked efficiency in service delivery [38]. In Pakistan, integration of polio, maternal and child health, and nutrition services through community mobilization, mobile outreach, and private-sector participation has previously improved vaccine acceptability and reach, highlighting the potential of private-sector engagement to address equity-related barriers [39,40,41]. Our antigen-wise analysis demonstrates similar trends and further substantiates the effectiveness of the PPE model, showing impactful and consistent gains across vaccine doses. The model had the greatest effect in bridging equity gaps for BCG and all first-dose vaccines (OPV-1, Rota-1, PCV-1, and Penta-1), while also producing more than a fourfold increase in odds for series-completion indicators (Penta-3, PCV-3, and OPV-3). These findings are noteworthy because high attrition due to dropouts between first and third doses are common in urban settlement populations due to mobility, informal employment, and fragmented health-seeking behavior [42]. Collectively, the improvements suggest that the PPE model not only facilitated vaccine initiation but also improved adherence to the full immunization schedule. Moreover, observed improvements in MR-1 and TCV, both administered after nine months of age, indicate that the intervention successfully reached older infants, a persistent challenge in routine immunization programs.

Although OPV-0 uptake increased slightly at endline, no statistically significant change was observed, likely due to already high baseline coverage attributable to ongoing mass polio campaigns [30,32,39,40,41]. Nevertheless, despite historically high dropout rates [30,41], coverage of subsequent OPV doses and other antigens improved markedly following implementation of the PPE model. In Pakistan and other LMICs, where nearly three-quarters of the population seek care from private providers [43], our findings indicates strategic engagement of the private sector can play a pivotal role in improving vaccine coverage by leveraging its proximity to the communities, resources, expertise, and community trust. Public–private engagement approaches have previously been implemented at scale through tuberculosis control programs in Pakistan and other countries, yielding encouraging implementation and coverage outcomes [44,45]. Extending such Engagements to immunization and other preventive health services therefore represents a promising strategy to mitigate access barriers and promote vaccine equity, particularly in urban settlements and underserved populations.

For future implementation, a multifaceted approach within the PPE framework is recommended to increase vaccine demand and address persistent coverage gaps in HRUCs and Super high risks Union Councils (SHRUCs). This includes culturally sensitive communication to counter misinformation, building trust through locally embedded private providers, and targeted outreach to mobile and migrant and minority populations such as Pashtos and Balochis. In Parallel, system-level reforms are required to institutionalize private-provider partnerships. Future studies should focus on optimizing the PPE model for scalability and evaluating its integration with other essential health services.

However, caution must be exercised as while the PPE model demonstrated strong effects in high-risk urban settlements, its scalability will depend on sustained financing, regulatory oversight, and integration within existing EPI delivery and reporting systems. The model leveraged existing private-sector infrastructure, potentially lowering capital costs; however, recurrent expenditures related to human resources, supervision, and community engagement must be considered for scale-up. Effectiveness may vary in non–high-risk urban or rural settings where private provider density, trust, and care-seeking patterns differ, requiring contextual adaptation and further implementation research.

Strengths and limitations: To our knowledge, this is the first study to evaluate the impact of engaging private providers on routine vaccination in urban settlements of Karachi. The results demonstrate a remarkable decline in zero-dose children.

However, generalizability may be limited, as the study was conducted in HRUCs of Karachi and may not represent patterns observed in less heterogeneous or less populous urban areas. Moreover, given the inherent limitation of the before–after quasi-experimental design and the absence of a parallel comparison arm, the observed improvements in immunization coverage cannot be attributed solely to the PPE intervention, as secular trends, ongoing mass immunization activities (including polio and catch-up campaigns), and broader system-level changes in Karachi’s immunization program during the study period may also have contributed to the observed gains.

In addition, the baseline and endline surveys sampled different participants residing in the same intervention areas; therefore, observed changes may reflect both temporal shifts in population characteristics and the effects of the intervention, limiting causal inference.

Finally, reliance on caregiver recall for vaccination status introduces potential recall bias. Larger multi-site studies are therefore needed to assess the replicability of these findings and to identify context-specific approaches, as no single approach is likely to be universally effective across diverse urban communities.

## 5. Conclusions

The findings suggest that engaging private providers led to increased overall immunization coverage and significant reductions in education- and occupation-related inequities. By leveraging neighborhood private providers, the PPE model likely addressed key implementation barriers, such as low health literacy, mistrust in public services, and physical distance to vaccination sites, that disproportionately affect marginalized households. Our results underscore the potential of the PPE model as a pro-equity implementation strategy to strengthen routine immunization systems and reduce the prevalence of zero-dose and unimmunized children in underserved and hard-to-reach urban populations.

## Figures and Tables

**Table 1:** Sociodemographic characteristics associated with vaccination status of children aged 4–11 Months.

Characteristics of Surveyed Mothers/Caregiver and Child	BL Total	Fully Immunized n (%)	Partially Immunized n (%)	Unvaccinated n (%)	EL Total	Fully Immunized n (%)	Partially Immunized n (%)	Unvaccinated n (%)
**Vaccination status**	1141	254 (22.26)	855 (74.93)	32 (2.80)	1026	527 (51.36)	490 (47.76)	9 (0.88)
**Sex of child**								
Male	565	137 (24.25)	410 (72.57)	18 (3.19)	523	267 (51.05)	250 (47.80)	6 (1.15)
Female	576	117 (20.31)	445 (77.26)	14 (2.43)	503	260 (51.69)	240 (47.71)	3 (0.60)
* Age	-	226.82 (64.21)	223.61 (67.76)	216.5 (69.13)	-	232.01 (67.57)	229.88 (68.65)	216.98 (66.98)
**Mother Education**								
No Education	728	97 (13.32)	603 (82.83)	28 (3.85)	610	261 (42.79)	341 (55.90)	8 (1.31)
Primary	81	22 (27.16)	57 (70.37)	2 (2.47)	157	103 (65.61)	53 (33.76)	1 (0.64)
Secondary and Higher	331	135 (40.79)	194 (58.61)	2 (0.60)	259	163 (62.93)	96 (37.07)	0 (0.00)
**Father Education**								
No Education	631	76 (12.04)	526 (83.36)	29 (4.60)	519	228 (43.93)	284 (54.72)	7 (1.35)
Primary	104	22 (21.15)	81 (77.88)	1 (0.96)	178	104 (58.43)	72 (40.45)	2 (1.12)
Secondary and Higher	405	155 (38.27)	248 (61.23)	2 (0.49)	329	195 (59.27)	134 (40.73)	0 (0.00)
**Mother Occupation**								
Unskilled	1107	246 (22.22)	829 (74.89)	32 (2.89)	941	482 (51.22)	451 (47.93)	8 (0.85)
Skilled	28	4 (14.29)	24 (85.71)	0 (0.00)	81	42 (51.85)	38 (46.91)	1 (1.23)
**Father Occupation**								
Unskilled	674	111 (16.47)	536 (79.53)	27 (4.01)	296	141 (47.64)	151 (51.01)	4 (1.35)
Skilled	467	143 (30.62)	319 (68.31)	5 (1.07)	725	383 (52.83)	337 (46.48)	5 (0.69)
**Ethnicity**								
Urdu	287	115 (40.07)	172 (59.93)	0 (0.00)	204	137 (67.16)	66 (32.35)	1 (0.49)
Balochi	31	6 (19.35)	23 (74.19)	2 (6.45)	35	18 (51.43)	16 (45.71)	1 (2.86)
Sindhi	126	30 (23.81)	93 (73.81)	3 (2.38)	150	66 (44.00)	83 (55.33)	1 (0.67)
Pushto	355	40 (11.27)	298 (83.94)	17 (4.79)	260	111 (43.19)	143 (55.64)	3 (1.17)
Punjabi	82	21 (25.61)	59 (71.95)	2 (2.44)	95	57 (60.00)	37 (38.95)	1 (1.05)
Saraiki	194	31 (15.98)	158 (81.44)	5 (2.58)	166	82 (49.40)	83 (50.00)	1 (0.60)
Other	66	11 (16.67)	52 (78.79)	3 (4.55)	116	54 (46.55)	61 (52.59)	1 (0.86)

* Mean (SD).

**Table 2:** Total antigen coverage and change in average coverage of vaccines between baseline and endline.

Type of Vaccine	BL n	BL % (95% CI)	EL n	EL % (95% CI)	Difference BL to EL % (95% CI)	Chi Square	*p*-Value
MR1	62	18.73 (14.67–23.35)	162	46.82 (41.46–52.23)	28.08 (21.35–34.82)	15.41	<0.001
OPV zero	1109	97.19 (96.06–98.07)	1010	98.44 (97.47–99.10)	1.24 (0.00–2.46)	3.87	<0.001
OPV-1	698	61.17 (58.27–64.01)	918	89.47 (87.43–91.28)	28.29 (24.90–31.69)	228.17	<0.001
OPV-2	514	45.05 (42.13–47.98)	811	79.04 (76.42–81.49)	33.99 (30.18–37.80)	262.81	<0.001
OPV-3	358	31.38 (28.69–34.15)	685	66.76 (63.78–69.64)	35.38 (31.44–39.33)	270.99	<0.001
BCG	972	85.18 (82.99–87.20)	1001	97.56 (96.42–98.41)	12.37 (10.10–14.64)	101.50	<0.001
Penta-1	724	63.45 (60.58–66.25)	920	89.67 (87.64–91.46)	26.21 (22.85–29.57)	202.77	<0.001
Penta-2	515	45.14 (42.22–48.07)	813	79.24 (76.62–81.68)	34.10 (30.29–37.91)	264.81	<0.001
Penta-3	364	31.90 (29.20–34.69)	685	66.76 (63.78–69.64)	34.86 (30.90–38.81)	262.90	<0.001
PCV-1	719	63.01 (60.13–65.82)	919	89.57 (87.53–91.37)	26.55 (23.18–29.92)	206.47	<0.001
PCV-2	511	44.79 (41.87–47.72)	812	79.14 (76.52–81.59)	34.35 (30.54–38.16)	268.18	<0.001
PCV-3	367	32.16 (29.45–34.96)	684	66.67 (63.68–69.54)	34.50 (30.54–38.45)	257.46	<0.001
Rota-1	722	63.28 (60.40–66.08)	919	89.57 (87.53–91.37)	26.29 (22.92–29.65)	203.18	<0.001
Rota-2	511	44.79 (41.87–47.72)	812	79.14 (76.52–81.59)	34.35 (30.54–38.16)	268.18	<0.001
IPV-1	386	33.83 (31.08–36.65)	685	66.76 (63.78–69.64)	32.93 (28.95–36.91)	234.41	<0.001
IPV-2	54	17.22 (13.31–21.72)	199	47.10 (41.75–52.51)	29.88 (23.24–36.53)	14.17	<0.001
TCV	62	18.73 (14.67–23.35)	162	46.82 (41.46–52.23)	28.08 (21.35–34.82)	12.02	<0.001

Abbreviations: BL = Baseline, EL = Endline, CI = Confidence Interval.

**Table 3:** Results of multinomial logistic regression for sociodemographic factors associated with immunization status.

	Fully Immunized			Partially Immunized		
	Univariate	Multivariate		Univariate	Multivariate	
	OR (95% CI)	OR (95% CI)	*p* Value	OR (95% CI)	OR (95% CI)	*p* Value
**Time**						
Baseline	1	1		1	1	
Endline	7.37 (1.96–27.64)	6.34 (2.45–16.21)	0.000	2.03 (0.69–5.94)	1.639 (0.64–4.14)	0.291
**Sex of child**						
Male	1	1		1	1	
Female	1.31 (0.53–3.24)	-		1.46 (0.59–3.59)	-	
**Age**	1.03 (0.99–1.08)	-		1.03 (0.99–1.08)	-	
**Mother Education**						
No Education	1	1		1	1	
Primary	4.18 (1.42–12.318)	1.32 (0.33–5.28)	0.690	1.39 (0.46–4.196)	0.72 (0.19–2.74)	0.632
Secondary and Higher	14.98 (5.55–40.398)	2.52 (0.70–9.18)	0.153	5.52 (1.79–16.99)	1.47 (0.36–5.87)	0.580
**Father Education**						
No Education	1	1		1	1	
Primary	4.97 (1.24–19.89)	2.15 (0.62–7.47)	0.222	2.26 (0.65–7.88)	1.74 (0.49–6.08)	0.380
Secondary and Higher	20.72 (6.05–70.92)	7.82 (2.09–29.24)	0.003	8.48 (2.80–25.68)	4.90 (1.41–17.06)	0.013
**Mother Occupation**						
Unskilled	1	1		1	1	
Skilled	2.52 (0.27–23.64)	-		1.93 (0.21–17.79)	-	
**Father Occupation**						
Unskilled	1	1		1	1	
Skilled	6.4 (2.68–15.58)	2.92 (1.38–6.18)	0.006	2.96 (1.47–5.95)	2.29 (1.09–4.81)	0.028
**Ethnicity**						
Urdu	1	1		1	1	
Balochi	0.03 (0.01–0.91)	0.07 (0.04–1.42)	0.083	0.055 (0.004–0.738)	0.14 (0.08–1.42)	0.089
Sindhi	0.09 (0.08–1.17)	0.21 (0.01–2.64)	0.225	0.185 (0.016–2.127)	0.34 (0.02–4.84)	0.427
Pushto	0.03 (0.02–0.37)	0.06 (0.05–0.82)	0.036	0.093 (0.009–0.935)	0.14 (0.01–1.90)	0.139
Punjabi	0.13 (0.05–2.01)	0.15 (0.09–2.44)	0.180	0.134 (0.008–2.251)	0.19 (0.01–3.33)	0.252
Siraiki	0.07 (0.04–1.45)	0.23 (0.01–3.51)	0.291	0.16 (0.01–2.19)	0.36 (0.02–5.15)	0.450
Other	0.06 (0.04–0.94)	0.08 (0.07–1.16)	0.060	0.11 (0.01–1.44)	0.17 (0.01–2.40)	0.190

Notes. Outcome for regression model was vaccination status as: fully Immunized, partially Immunized and unvaccinated, and are presented as unadjusted and adjusted odds ratios (95% confidence Intervals) with reference to the unvaccinated category. Multivariable outcomes with *p* values smaller than 0.05 appear in bold font. Abbreviations: OR = odds ratio, CI = Confidence Interval, 1 = Reference. *p* value of both adjusted models: 0.000.

## Data Availability

Data is available on request from the corresponding author.

## Supplementary Materials

The following supporting information can be downloaded at: https://www.mdpi.com/article/10.3390/vaccines14030205/s1, Figure S1: Project Design; Table S1: Table S1a: Binary Logistic Regression Results for Age-Appropriate Antigen Uptake from Baseline to Endline Among Children Aged 4–11 Months; Table S1b: Variance Inflation Factors (VIF) Assessing Multicollinearity; Table S1c: Results of multinomial logistic regression for sociodemographic factors associated with immunization status among children without vaccination card (recall); Table S1d: Results of multinomial logistic regression for sociodemographic factors associated with immunization status among children with vaccination card.

## Abbreviations

The following abbreviations are used in this manuscript:

GAVI	Global Alliance for Vaccines and Immunization
WHO	World Health Organization
PPE	Private Provider Engagement
HRUC	High-Risk Union Council
