# Supplementary material for: Strengthening the Immunization System Through Private Provider Engagement to Improve Vaccine Uptake in Urban Settlements of Karachi, Pakistan: A Before–After Study
Source: Vaccines (Basel). 2026 Feb 26;14(3):205. doi: 10.3390/vaccines14030205 (PMC13030395; doi:10.3390/vaccines14030205)
Supplement: Supplementary file 1 [file vaccines-14-00205-s001.zip › vaccines-4102579-supplementary.pdf]

Supplementary Figure S1: Project Design

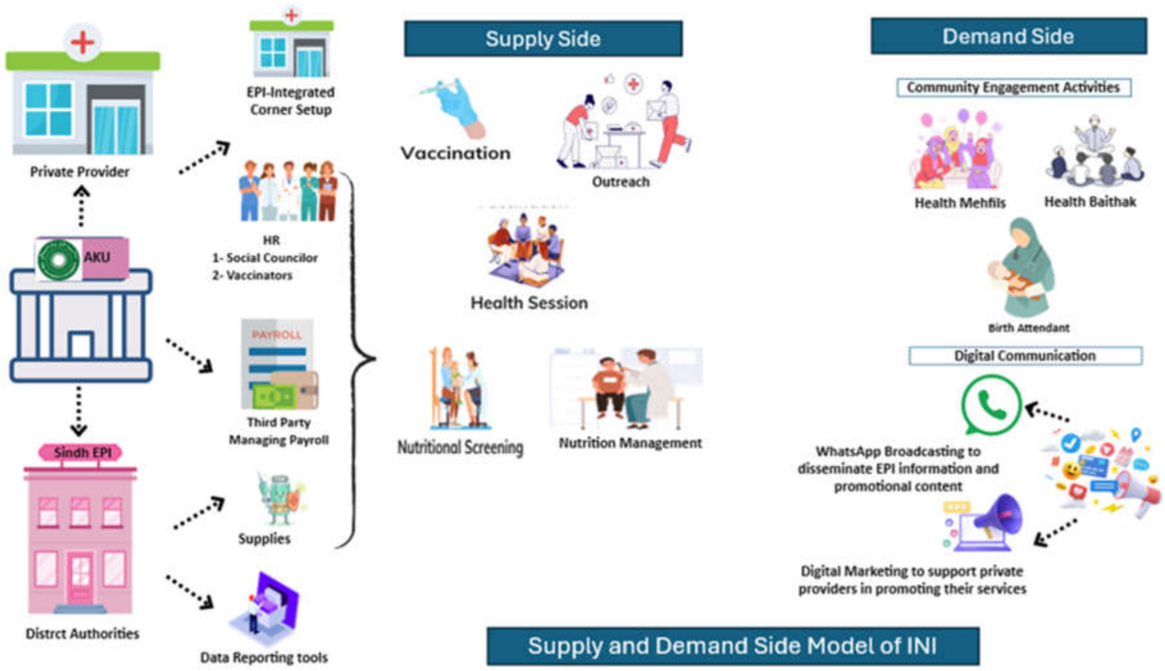

Supplementary Table S1a: Binary Logistic Regression Results for Age-Appropriate Antigen Uptake from Baseline to Endline Among Children Aged 4–11 Months.

Age-appropriate vaccines	Unadjusted (OR)<br>OR (95% CI)	Adjusted (OR)<br>OR (95% CI)
At Birth		
BCG	6.96 (2.38–20.31)	6.47 (2.63–15.92)
OPV0	1.82 (0.68–5.45)	1.28 (0.55–2.97)
6 Weeks (2nd Visit)		
OPV-1	5.39 (3.15–9.21)	5.64 (3.92–8.12)
Pentavalent 1	4.99 (2.93–8.53)	5.12 (3.48–7.54)
PCV-1	5.04 (2.93–8.64)	5.28 (3.51–7.74)
Rotavirus 1	4.98 (2.89–8.57)	5.15 (3.49–7.59)
10 Weeks (3rd Visit)		
OPV-2	4.60 (2.84–7.43)	4.95 (3.62–6.77)
Pentavalent 2	4.63 (2.88–7.45)	4.93 (3.61–6.75)
PCV-2	4.67 (2.90–7.542)	5.01 (3.63–6.91)
Rotavirus 2	4.67 (2.92–7.49)	4.98 (3.65–6.79)
14 Weeks (4th Visit)		
OPV-3	4.39 (2.66–7.24)	4.83 (3.72-–6.28)
Pentavalent 3	4.28 (2.67–6.87)	4.55 (3.55–5.82)
PCV-3	4.21 (2.61–6.79)	4.46 (3.48–5.71)
IPV-1	3.92 (2.43–6.34)	4.08 (3.25–5.21)
9 Months (5th Visit)		
MR-1	1.52 (1.17–2.08)	3.67 (2.37–5.67)
IPV-2	1.49 (1.13–2.02)	3.66 (2.39–5.59)
TCV	1.44 (1.06–1.96)	3.44 (2.27–5.21)

Notes: a. Binary logistic regression was conducted separately for each antigen, treated as an individual outcome, and is presented as unadjusted and adjusted odds ratios (95% confidence intervals) with reference category for each antigen as baseline

b. Adjusted models controlled for age of the child, sex of the child, mother's education, father's education, mother's occupation, father's occupation, and ethnicity

Abbreviations: OR=odds ratio, CI=Confidence Interval

p-value of adjusted model: <0.001

Supplementary Table S1b: Variance Inflation Factors (VIF) Assessing Multicollinearity

Variable	VIF	Tolerance (1/VIF)
Mother Education	1.03	0.969
Primary	1.26	0.791
Secondary & Higher	1.79	0.558
Ethnicity		
Balochi	1.23	0.814
Sindhi	2.19	0.456
Pushto	1.37	0.731
Punjabi	2.13	0.469
Siraiki	1.68	0.594
Other	1.28	0.782
Father Education		
Primary	1.25	0.797
Secondary & Higher	1.66	0.602
Father Occupation	1.18	0.851
Sex of child	1.00	0.996
Mean VIF	1.47	-

Notes: All VIF < 3; highest = 2.19. No multicollinearity seen

Supplementary Table S1c: Results of multinomial logistic regression for sociodemographic factors associated with immunization status among children without vaccination card (recall)

Variable	Fully Immunized		Partially Immunized	
	**Multivariate RRR (95% CI)**	**p-value**	**Multivariate RRR (95% CI)**	**p-value**
Time				
Baseline	Reference	-	Reference	-
Endline	6.27 (2.23–17.62)	0.001	1.42 (0.53–3.81)	0.477
Mother Education		-		-
No education	Reference	-	Reference	-
Primary	1.20 (0.31–4.62)	0.793	0.70 (0.19–2.58)	0.586
Secondary & Higher	2.28 (0.61–8.54)	0.216	1.34 (0.33–5.43)	0.681
Father Education				
No education	Reference	-	Reference	-
Primary	2.13 (0.60–7.63)	0.240	1.76 (0.49–6.28)	0.378
Secondary & Higher	7.27 (1.84–28.80)	0.005	5.05 (1.36–18.82)	0.017
Father Occupation		-		-
Unskilled	Reference	-	Reference	-
Skilled	2.41 (1.15–5.05)	0.020	1.98 (0.94–4.15)	0.072
Ethnicity				
Urdu	Reference	-	Reference	-
Baluchi	0.12 (0.01–1.85)	0.126	0.17 (0.01–2.10)	0.164
Sindhi	0.26 (0.02–3.15)	0.285	0.39 (0.03–4.90)	0.460
Pushto/Hindko	0.07 (0.01–0.91)	0.042	0.16 (0.01–1.97)	0.151
Punjabi	0.16 (0.01–2.51)	0.190	0.18 (0.01–2.85)	0.220
Siraiki	0.23 (0.02–3.11)	0.264	0.35 (0.03–4.51)	0.418
Other	0.07 (0.01–0.76)	0.029	0.13 (0.01–1.52)	0.102

Supplementary Table S1d: Results of multinomial logistic regression for sociodemographic factors associated with immunization status among children with vaccination card

Variable	Fully Immunized		Partially Immunized	
	**Multivariate RRR (95% CI)**	**p-value**	**Multivariate RRR (95% CI)**	**p-value**
Time				
Baseline	Reference	-	Reference	-
Endline	7.56 (2.84–20.14)	<0.001	2.05 (0.82–5.11)	0.120
Mother Education				
No education	Reference	-	Reference	-
Primary	1.31 (0.35–4.87)	0.685	0.79 (0.22–2.86)	0.714
Secondary & Higher	1.33 (0.49–3.61)	0.566	0.87 (0.31–2.40)	0.778
Father Education				
No education	Reference	-	Reference	-
Primary	2.38 (0.65–8.72)	0.188	1.96 (0.53–7.20)	0.307
Secondary & Higher	6.18 (1.91–20.00)	0.003	4.38 (1.43–13.43)	0.010
Father Occupation				
Unskilled	Reference	-	Reference	-
Skilled	2.87 (1.41–5.82)	0.004	2.30 (1.14–4.65)	0.021
Ethnicity				
Urdu	Reference	-	Reference	-

Baluchi	0.08 (0.01–1.11)	0.060	0.10 (0.01–1.17)	0.066
Sindhi	0.20 (0.02–2.38)	0.199	0.33 (0.03–4.06)	0.379
Pushto/Hindko	0.06 (0.01–0.77)	0.031	0.14 (0.01–1.66)	0.117
Punjabi	0.07 (0.01–0.91)	0.042	0.09 (0.01–1.24)	0.072
Siraiki	0.16 (0.01–2.11)	0.162	0.28 (0.02–3.46)	0.314
Other	0.05 (0.00–0.52)	0.013	0.10 (0.01–1.20)	0.069
